# Cognitive control in honesty and dishonesty under different conflict scenarios: insights from reaction time

**DOI:** 10.3389/fpsyg.2024.1271916

**Published:** 2024-03-14

**Authors:** Hao-Ming Li, Wen-Jing Yan, Yu-Wei Wu, Zi-Ye Huang

**Affiliations:** ^1^Wenzhou Seventh People’s Hospital, Wenzhou, China; ^2^School of Mental Health, Wenzhou Medical University, Wenzhou, China; ^3^Student Affairs Division, Wenzhou Business College, Wenzhou, China

**Keywords:** honesty, dishonesty, cognitive control, moral decision-making, reaction time

## Abstract

This study investigated the role of cognitive control in moral decision-making, focusing on conflicts between financial temptations and the integrity of honesty. We employed a perceptual task by asking participants to identify which side of the diagonal contained more red dots within a square to provoke both honest and dishonest behaviors, tracking their reaction times (RTs). Participants encountered situations with no conflict, ambiguous conflict, and clear conflict. Their behaviors in the clear conflict condition categorized them as either “honest” or “dishonest.” Our findings suggested that, in ambiguous conflict situations, honest individuals had significantly longer RTs and fewer self-interest responses than their dishonest counterparts, suggesting a greater need for cognitive control to resolve conflicts and a lesser tendency toward self-interest. Moreover, a negative correlation was found between participants’ number of self-interest responses and RTs in ambiguous conflict situations (*r* = −0.27 in study 1 and *r* = −0.66 in study 2), and a positive correlation with cheating numbers in clear conflict situations (*r* = 0.36 in study 1 and *r* = 0.82 in study 2). This suggests less cognitive control was required for self-interest and cheating responses, bolstering the “Will” hypothesis. We also found that a person’s self-interest tendency could predict their dishonest behavior. These insights extend our understanding of the role of cognitive control plays in honesty and dishonesty, with potential applications in education, policy-making, and business ethics.

## 1 Introduction

Human behavior is often governed by complex decision-making processes, with one recurring challenge being the conflict between self-interest and the pursuit of moral righteousness. This moral quandary, the struggle between the temptation of personal financial gain and the aspiration to uphold an honest image, unfolds in various scenarios ranging from relatively minor instances of tax evasion and inflated expense reports, to more severe instances of fraudulent financial schemes (Mazar et al., 2008). Such moral dilemmas offer a fascinating window into human behavior and motivations. They invite questions regarding how individuals reconcile these seemingly incompatible drives of personal gain and moral obligation. An increasingly explored proposition within the behavioral sciences is that cognitive control, our inherent ability to regulate thoughts, emotions, and actions, acts as a mediator in this tension between self-interest and moral self-image (Wood et al., 2008).

Despite its intuitive appeal, the role of cognitive control in moral decision-making, particularly its contribution to resolving conflicts between self-interest and honesty, remains a contentious topic within psychological and neuroscientific research. Although a large amount of data is available, the results are mixed (Köbis et al., 2019; Capraro, 2023). This debate predominantly centers around two main hypotheses: the “Will” hypothesis and the “Grace” hypothesis (Tabatabaeian et al., 2015). The “Will” hypothesis paints a less flattering image of human nature. It posits that humans, by default, are selfish and dishonest, and that it is cognitive control that keeps these basic instincts in check, compelling individuals toward honesty (Gino et al., 2011). This hypothesis aligns with traditional economic models of human behavior, which suggest that individuals are naturally driven to pursue self-interest, with social norms, laws, and moral values acting as external constraints on these inborn desires (Becker, 1968; Henrich et al., 2005). Contrastingly, the “Grace” hypothesis presents a more favorable image of humans, suggesting that people are essentially honest, and that cognitive control is used to suppress instinctual honest responses when there are opportunities to profit from dishonesty (Rand et al., 2012). This view is supported by empirical research that shows individuals respond faster when instructed to tell the truth than when directed to lie, suggesting honesty may indeed be more intuitive (Capraro, 2017; Suchotzki et al., 2017; Verschuere et al., 2018; Capraro et al., 2019).

This ongoing debate is far from a mere academic exercise. Instead, it underscores the complex, multifaceted nature of human morality, highlighting the need for more nuanced and empirical investigations into the interplay between cognitive control and moral behavior (Baumeister et al., 2009). As Baumeister and Exline (1999) propose, understanding these moral dynamics requires acknowledging individual differences, considering situational variables, and appreciating the dynamic nature of moral decision-making processes. Study found that the social consequences of lying could be a promising key to the riddle of intuition’s role in honesty. When dishonesty harms abstract others, promoting intuition causes more people to lie and people to lie more. However, when dishonesty harms concrete others, promoting intuition has no significant effect on dishonesty (Köbis et al., 2019). Recent research advancements have further complicated this landscape. With the advent of neuroimaging techniques, studies suggest that the impact of cognitive control on moral behavior may be dependent on an individual’s inherent moral disposition toward honesty or dishonesty (Greene and Paxton, 2009). Specifically, brain regions associated with cognitive control, such as the anterior cingulate cortex and the inferior frontal gyrus, have been found to help individuals predisposed toward dishonesty to act honestly, while enabling those predisposed toward honesty to cheat when the situation permit (Speer et al., 2022).

Against this backdrop, the present study embarks on an exploration of how individuals, predisposed toward honesty or dishonesty, respond to situations that present a conflict between personal financial gain and moral self-image. Beyond neuroimaging, reaction time (RT) measures, often utilized in cognitive psychology, are believed to offer critical insights into cognitive control’s involvement in moral decision-making conflicts (Evans et al., 2015; Andrighetto et al., 2020). RTs provide non-invasive, real-time evidence of the cognitive processes at play during moral decision-making (Shalvi et al., 2012). This study introduces three distinct decision conflict scenarios, allowing for a more nuanced examination of individual differences in cognitive control and moral tendencies. By analyzing the interaction between cognitive control, moral inclination, and response times across these scenarios, we hope to provide a more comprehensive, more dynamic, and ultimately, a more human perspective on the landscape of moral decision-making (Tangney et al., 2007).

## 2 Study 1

### 2.1 Method

#### 2.1.1 Participants

We recruited sixty-seven undergraduate or postgraduate students from Wenzhou University. The participants, with an average age of 19.39 (*SD* = 1.18), comprised 39 females and 28 males. All participants had normal or corrected-to-normal vision and provided informed consent before the study. The study adhered to the sixth revision of the Declaration of Helsinki (2008) and was approved by the university’s Institutional Review Board (IRB).

#### 2.1.2 Procedure

We engaged the participants in a perceptual task (Gino et al., 2010). Each trial presented a square divided diagonally into two sections. Each section had 20 dots scattered randomly on the left or right side of the diagonal. After a one-second exposure, participants identified which side of the diagonal held more dots by clicking the respective mouse button. The reward for each trial was calculated as follows: clicking the left mouse button yielded 0.02CNY, whereas clicking the right button yielded 0.2CNY. Therefore, trials with more dots on the left side of the diagonal presented a conflict between answering accurately and maximizing profit.

The perceptual task was split into two phases. The first phase consisted of 100 practice trials, after which participants received feedback on potential earnings for each trial and cumulative earnings if these trials had involved real payment. In the second phase, the participants completed 200 trials, earning real money, and received information about their earnings for each trial and overall.

Participants could earn a maximum of 40CNY on this perceptual task (by always pressing the right mouse button). There were four blocks. Each block consisted of 50 trials, and each block included 8 trials in which the answer was clearly “more on right” (no conflict condition, i.e., the ratio of the number of dots on the right to the number of dots on the left was greater than or equal to 1.5), 17 trials in which the answer was clearly “more on left” (clear conflict condition, i.e., the ratio of the number of dots on the right to the number of dots on the left was less than or equal to 2/3), and 25 ambiguous trials (ambiguous conflict condition, i.e., the ratio of the number of dots on the right to the number of dots on the left was between 2/3 and 1.5). The responses in ambiguous condition reflect an individual’s self-interest tendency. Once participants completed this task, the computer indicated that they should report their performance in Phase 2 on a collection slip to be handed to the experimenter at the end of the study.

### 2.2 Results

**Figure 1:**
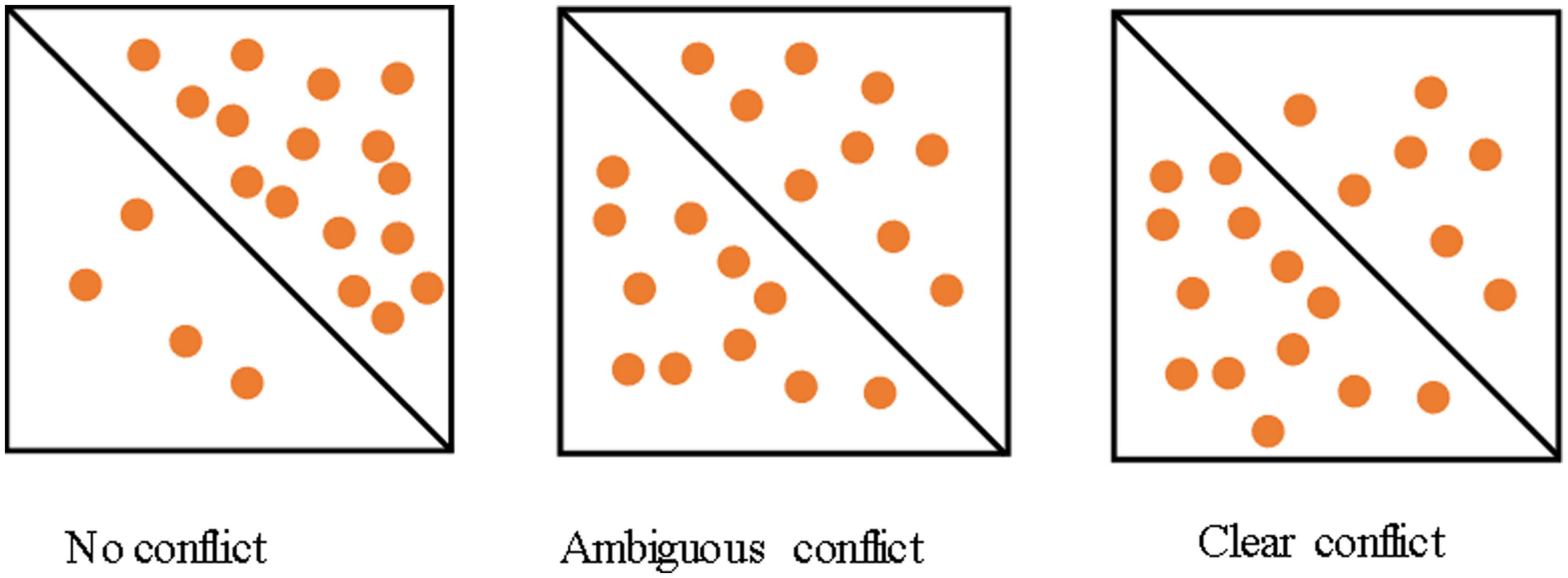
Examples of decision conflicts.

All participants displayed honest behavior in no conflict condition. A total of 53.73% (36/67) participants were found to cheat one or more times in clear conflict condition (see Figure 1). The participants who cheated in clear conflict condition (36 participants, mean cheating number = 23.63) will be referred to as ‘dishonest individuals’, while the remaining participants (31 participants) will be referred to as ‘honest individuals’.

We compared the reaction times (RTs) in the no conflict, ambiguous conflict and clear conflict conditions among honest and dishonest participants. Results showed that honest individuals required longer RTs than dishonest individuals in the ambiguous conflict condition, *p* = 0.047, suggesting that honest individuals required more time to revolve ambiguous conflict. Also, honest individuals made less self-interest responses (*M* = 42, *SD* = 5.08) than dishonest individuals (*M* = 67.42, *SD* = 22.34) in the ambiguous condition, *p* < 0.001. There were no RT differences in no conflict and clear conflict conditions among honest and dishonest participants, *p* = 0.07; *p* = 0.09.

Moreover, the RTs in ambiguous trials correlated with the self-interest numbers in the ambiguous condition, *r* = −0.27, *p* = 0.028 and the cheating numbers in the clear conflict condition, *r* = −0.24, *p* = 0.046. The self-interest numbers in the ambiguous condition correlated with the cheating numbers in the clear conflict condition, *r* = 0.36, *p* = 0.003. When using RTs and self-interest numbers in the ambiguous condition to predict the cheating numbers in the clear conflict condition, the model was significant, with R2 = 0.15, *p* = 0.005. The self-interest number in the ambiguous condition was a significant indicator of cheating numbers in the clear conflict condition, *p* = 0.01; whereas the RTs in the ambiguous conditions was not significant in the model, *p* = 0.19.

## 3 Study 2

### 3.1 Method

#### 3.1.1 Participant

We recruited ninety-five undergraduate or postgraduate students from Hebei Normal University. The participants, with an average age of 19.55 (*SD* = 1.07), comprised 75 females and 20 males. All participants had normal or corrected-to-normal vision and provided informed consent before the study. The study adhered to the sixth revision of the Declaration of Helsinki (2008) and was approved by the university’s Institutional Review Board (IRB).

#### 3.1.2 Procedure

The task is same as that of Study 1, only some differences in experimental materials. In the experiment, 18 images were made in the order of the left and right red dots from less to most. The experiment consisted of 4 blocks, and 18 images in each block were randomly presented 4 times, for a total of 72 trials. The experiment consisted of a total of 288 trials.

### 3.2 Results

All participants displayed honest behavior in no conflict condition. A total of 54.74% (52/95) participants were found to cheat one or more times in clear conflict condition. The participants who cheated in clear conflict condition (52 participants, mean cheating number = 29.98) will be referred to as ‘dishonest individuals’, while the remaining participants (43 participants) will be referred to as ‘honest individuals’.

We compared the reaction times (RTs) in the no conflict, ambiguous conflict and clear conflict conditions among honest and dishonest participants. Results showed that honest individuals required longer RTs (*M* = 665.25, *SD* = 143.04) than dishonest individuals in (*M* = 550.64, *SD* = 166.12) the ambiguous conflict condition, *p* = 0.001, suggesting that honest individuals required more time to revolve ambiguous conflict. Also, honest individuals made less self-interest responses (*M* = 3.50, *SD* = 2.48) than dishonest individuals (*M* = 9.69, *SD* = 4.78) in the ambiguous condition, *p* < 0.001. Moreover, the RTs in ambiguous trials correlated with the self-interest numbers in the ambiguous condition, *r* = −0.66, *p* < 0.001 and the cheating numbers in the clear conflict condition, *r* = −0.65, *p* < 0.001. The self-interest numbers in the ambiguous condition correlated with the cheating numbers in the clear conflict condition, *r* = 0.82, *p* < 0.001. When using RTs and self-interest numbers in the ambiguous condition to predict the cheating numbers in the clear conflict condition, the model was significant, with R^2^ = 0.73, *p* < 0.001. The self-interest number and the RTs in the ambiguous conditions were significant indicators of cheating numbers in the clear conflict condition, *p* < 0.001; *p* = 0.003.

We also investigated the effect of conflict degree on the RTs of honest and dishonest people. Subtract the non-conflicting RTs from the conflicting RTs corresponding to the left and right red dots (i.e., the RTs under the condition that the left red dot is 13 minus the RTs under the condition that the right red dot is 7; The RTs under the condition that the left red dot is 14 minus the RTs under the condition that the red dot on the right is 6; and so on). We believe that the smaller difference between the numbers of red dots on the left and right, the greater psychological conflict of the individual. The results showed that conflict degree affected the participants’ responses, the greater the conflict, the longer RTs required, *F*(6, 498) = 47.67, *p* < 0.001. There was no difference between the honest and dishonest people in their RTs at different conflict levels, *p* = 0.46.

## 4 Discussion

Our study investigated the interplay between cognitive control and moral decision-making, particularly focusing on how individuals with different predispositions toward honesty or dishonesty react in situations where personal financial gain conflicts with moral self-image. The key finding is that individuals who are inherently more honest exhibited longer reaction times in scenarios with ambiguous moral conflicts, suggesting a deeper cognitive engagement in these dilemmas. Conversely, those predisposed to dishonesty responded more quickly, implying less cognitive deliberation. This differentiation highlights the complex role of cognitive control in navigating moral decisions, indicating that it is influenced by an individual’s moral inclinations. Essentially, our results contribute to understanding the nuanced mechanisms behind moral behavior, showing that moral decision-making is a dynamic process shaped by both cognitive control and personal ethical standards.

Our observation that honest individuals exhibit longer reaction times in ambiguous conflict conditions than their dishonest counterparts offers an intriguing insight into the cognitive processes underlying moral behavior. This finding aligns with the work of Capraro and Rand (2018), who suggested that honesty might be more intuitive to individuals with a stronger predisposition toward prosocial behavior, requiring less cognitive control in clear-cut situations but more deliberation when the context is ambiguous. Our results extend this theory by quantitatively showing that the cognitive effort, as measured by reaction times, increases in moral dilemmas where the right choice is not immediately apparent.

Additionally, the correlation between reaction times and self-interest behaviors in ambiguous and clear conflict conditions, as observed in our study, indicates a dynamic interplay between cognitive control and situational factors. This extends the findings of Shalvi et al. (2012), who highlighted the role of situational clarity in ethical decision-making. Our results further elaborate on this by showing that the ambiguity of a situation not only affects decision-making speed but also interacts with an individual’s moral inclination to influence their choices.

Furthermore, our study contributes to the debate surrounding the “Will” and “Grace” hypotheses. The negative correlation between cognitive control and the number of self-interest responses suggests that honesty, far from being the default human condition, may be the product of a conscious cognitive effort to restrain self-serving impulses. This would be consistent with the “Will” hypothesis.

### 4.1 Applications and limitations

The results extend our understanding of the role of cognitive control plays in honesty and dishonesty, with potential applications in education, policy-making, and business ethics. For educational settings, the results suggest curricula should emphasize enhancing ethical reasoning and cognitive control, preparing students to navigate moral challenges thoughtfully. Policy implications include designing environments that discourage dishonesty by clarifying ethical standards and making dishonest actions more cognitively taxing, thereby promoting transparency and accountability. In business ethics, our findings advocate for cultures of integrity supported by clear ethical guidelines and training programs that bolster moral awareness and cognitive control, helping employees prioritize ethical standards over self-interest. This approach aims to foster a more honest and ethical conduct across various sectors.

Our study, while offering valuable insights into the complex interplay between cognitive control and moral decision-making, is not without its limitations. One of the primary constraints involves the sample size and demographic composition, primarily undergraduate and postgraduate students, which may not fully represent the broader population. This limitation could affect the generalizability of our findings, as the specific age group and educational background of our participants might influence their moral decision-making processes and cognitive control mechanisms differently compared to a more diverse population. Additionally, our reliance on reaction times as the use of intuitive or reflective processes should be careful. Rather some studies highlight the pitfalls of using RT correlations as support for dual-process theories. Reaction times, in this context, primarily reflect the cognitive processing involved in navigating moral conflicts rather than directly indicating whether honesty is an inherent or automatic response (Evans et al., 2015; Krajbich et al., 2015; Andrighetto et al., 2020).

## 5 Conclusion

Our study contributes to the nuanced understanding of the interplay between cognitive control and moral decision-making, revealing the complex mechanisms through which individuals navigate ethical dilemmas. By examining the roles of decision conflict and moral deliberation across different moral predispositions, our findings challenge and extend existing theories on moral psychology. Despite limitations related to sample diversity and the interpretation of reaction times, this research underscores the importance of considering individual differences and the multifaceted nature of cognitive processes in ethical behavior. Looking forward, it paves the way for further interdisciplinary investigations into moral decision-making, encouraging a broader exploration of how cognitive, emotional, and social factors collectively shape our moral actions. As we continue to unravel the cognitive underpinnings of morality, this work not only deepens our theoretical understanding but also has practical implications for promoting ethical behavior in an increasingly complex world.

## Data availability statement

The raw data supporting the conclusions of this article will be made available by the authors, without undue reservation.

## Ethics statement

The studies involving humans were approved by IRB of the Seventh People’s Hospital of Wenzhou (EC-KY-2022048). The studies were conducted in accordance with the local legislation and institutional requirements. The participants provided their written informed consent to participate in this study.

## Author contributions

H-ML: Writing – original draft. W-JY: Conceptualization, Writing – original draft. Y-WW: Conceptualization, Writing – original draft. Z-YH: Writing – review & editing, Conceptualization, Investigation, Validation.
